# Central role of the flowering repressor *ZCCT2* in the redox control of freezing tolerance and the initial development of flower primordia in wheat

**DOI:** 10.1186/1471-2229-14-91

**Published:** 2014-04-07

**Authors:** Zsolt Gulyás, Ákos Boldizsár, Aliz Novák, Gabriella Szalai, Magda Pál, Gábor Galiba, Gábor Kocsy

**Affiliations:** 1 Agricultural Institute, Centre for Agricultural Research, Hungarian Academy of Sciences, Brunszvik u. 2, 2462 Martonvásár, Hungary; 2 Doctoral School of Molecular and Nanotechnologies, Research Institute of Chemical and Process Engineering, Faculty of Information Technology, University of Pannonia, Egyetem u. 10, 8200 Veszprém, Hungary; 3 Doctoral School of Animal and Agricultural Environmental Sciences, Department of Plant Sciences and Biotechnology, Georgikon Faculty, University of Pannonia, Deák Ferenc u. 16, 8360 Keszthely, Hungary

**Keywords:** Glutathione, Redox state, Initial development of flower primordia, Freezing tolerance, Wheat, *ZCCT2* gene

## Abstract

**Background:**

As both abiotic stress response and development are under redox control, it was hypothesised that the pharmacological modification of the redox environment would affect the initial development of flower primordia and freezing tolerance in wheat (*Triticum aestivum L.*).

**Results:**

Pharmacologically induced redox changes were monitored in winter (*T. ae.* ssp*. aestivum* cv. Cheyenne, Ch) and spring (*T. ae.* ssp*. spelta*; Tsp) wheat genotypes grown after germination at 20/17°C for 9 d (chemical treatment: last 3 d), then at 5°C for 21 d (chemical treatment: first 4 d) and subsequently at 20/17°C for 21 d (recovery period). Thiols and their disulphide forms were measured and based on these data reduction potentials were calculated. In the freezing-tolerant Ch the chemical treatments generally increased both the amount of thiol disulphides and the reduction potential after 3 days at 20/17°C. In the freezing-sensitive Tsp a similar effect of the chemicals on these parameters was only observed after the continuation of the treatments for 4 days at 5°C. The applied chemicals slightly decreased root fresh weight and increased freezing tolerance in Ch, whereas they increased shoot fresh weight in Tsp after 4 days at 5°C. As shown after the 3-week recovery at 20/17°C, the initial development of flower primordia was accelerated in Tsp, whereas it was not affected by the treatments in Ch. The chemicals differently affected the expression of *ZCCT2* and that of several other genes related to freezing tolerance and initial development of flower primordia in Ch and Tsp after 4 d at 5°C.

**Conclusions:**

Various redox-altering compounds and osmotica had differential effects on glutathione disulphide content and reduction potential, and consequently on the expression of the flowering repressor *ZCCT2* in the winter wheat Ch and the spring wheat Tsp. We propose that the higher expression of *ZCCT2* in Ch may be associated with activation of genes of cold acclimation and its lower expression in Tsp with the induction of genes accelerating initial development of flower primordia. In addition, *ZCCT2* may be involved in the coordinated control of the two processes.

## Background

Throughout their life cycle plants are affected by various abiotic stresses, such as drought, extreme temperature, high salt concentration and cold, and these cause notable yield reductions in agriculture worldwide. The genetically determined level of freezing tolerance is achieved during cold acclimation, which is a relatively slow, adaptive response during autumn, when the temperature, day length and light intensity usually decrease gradually [1]. Two main signalling pathways ensure the reprogramming of the plant metabolism in *Arabidopsis* during this process; one is dependent on abscisic acid (ABA), whereas the other is not [2]. In the ABA-independent pathway the C-REPEAT BINDING FACTOR/DEHYDRATION-RESPONSIVE ELEMENT BINDING FACTOR (CBF/DREB1) plays a central role both in *Arabidopsis* and in crop species, including wheat (*Triticum aestivum* L.) and barley (*Hordeum vulgare* L.) [3]. At least 11 different *CBF* gene-coding sequences were mapped at the *Fr-2* locus of chromosome 5A in wheat, and *CBF14* has been found to be one of the most effective ones in increasing freezing tolerance both in wheat and barley [4-6]. *CBF*s are characterized by a plant-specific APETALA2/ETHYLENE-RESPONSIVE ELEMENT BINDING domain (AP2/ERF) [7,8], which interacts with the C-repeat elements present in the promoter region of their target genes. These are *COLD-REGULATED* (*COR*) genes making up the *CBF* regulon, the activation of which increases freezing tolerance. One of these genes, *COR14b,* is well characterized in barley and wheat [9,10]. It is differentially expressed in freezing-sensitive and freezing-tolerant genotypes, and helps to protect the photosynthetic apparatus from photo-oxidative damage during exposure to high-intensity light at freezing temperatures.

The decreasing temperature during autumn also fulfils the vernalization requirement of winter cereals and ensures the correct timing of the vegetative/generative transition and the protection of freezing-sensitive flowers [11]. In contrast, spring cereals do not require any cold treatment to induce flowering. Allelic differences in the main wheat *VERNALIZATION* genes *VRN1*, *VRN2* and *VRN3* determine the timing of the transition from vegetative to reproductive development. The MADS-box transcription factor VRN1 promotes flowering by inhibiting genes in the *VRN2* locus [12,13]. The *VRN2* locus contains two genes, *ZCCT1* and *ZCCT2* (encoding ZINC-FINGER/CONSTANS, CONSTANS-LIKE, TOC1 domain transcription factors) that are both involved in flowering repression [11]. *VRN3* encodes a RAF kinase inhibitor-like protein that displays a high degree of sequence identity to *Arabidopsis* FLOWERING LOCUS T (FT) protein [14]. The FT protein is a long-distance flowering signal that moves from the leaves to the apices through the phloem and promotes flowering [15]. The interactions between these three genes and their possible effect on freezing tolerance have been recently reviewed [11,16].

The coordinated regulation of vernalization and cold acclimation has been demonstrated in wheat, since *VRN1* allelic variation influences the duration of the expression of low temperature-induced genes [17]. In particular, mutations in the *VRN1* promoter, resulting in high *VRN1* transcript levels under both long and short days dampen the expression of the *COR* genes and lower freezing tolerance, especially under long-day conditions [16,18]. In addition, maximum freezing tolerance usually coincides with vernalization saturation in barley [19]. Thus, the hypothesis of *VRN1* pleiotropy would explain the fact, long known to breeders, that winter-type genotypes of wheat and barley carrying a vernalization-sensitive (“winter”) allele at the *VRN1* locus are more freezing-tolerant than spring-type cultivars. Another link between the regulation of vernalization and the stress response exists through the NUCLEAR FACTOR Y complex (NF-Y) consisting of A, B and C subunits. An interaction between NF-YB and ZCCT (VRN2) proteins has been detected in wheat [20], and NF-Y has also proved to be involved in tolerance to abiotic stress in *Arabidopsis*[21]. The NF-Y complex may affect the stress response through its interaction with the bZIP proteins controlling ABA signalling, as shown in *Arabidopsis*[22].

Freezing tolerance and initial development of flower primordia, like many adaptive and developmental processes, are under redox control in plants [23]. Unfavourable environmental conditions induce oxidative stress [24]. Reactive oxygen species (ROS), such as superoxide radicals, hydrogen peroxide, hydroxyl radicals and singlet oxygen may accumulate to toxic levels, leading to serious injury or plant death because of redox imbalance [25]. However, a moderate increase in the ROS level may activate various defence mechanisms through redox signalling pathways [26,27]. The enzymatic and non-enzymatic compounds in the antioxidant system may be affected, including ascorbate and glutathione, which are the heart of the redox hub [28].

Alterations in ROS and antioxidant levels are not only induced by various environmental effects, but may also occur during the growth and development of plants. Tissue-, cell- and compartment-specific spatial and temporal variations in their levels are especially important. One of the most important antioxidants is glutathione [glutathione was used generically in this paper to indicate reduced glutathione (GSH) and glutathione disulphide (GSSG)], which is a multifunctional metabolite that interacts with several molecules through thiol-disulphide exchange and de-glutathionylation and also participates in detoxification, defence, metabolism, redox signalling and the regulation of transcription and protein activity [26,29]. Changes in the amount and ratio of GSH and GSSG affect cellular reducing capacity and half-cell reduction potential, which can be used as stress markers [30,31]. The biosynthesis of GSH was stimulated by low temperature in wheat, and this change was greater in freezing-tolerant genotypes than in sensitive ones [32]. After 3 weeks of cold treatment there was a correlation between the H_2_O_2_, ascorbate and glutathione contents, the ascorbate/dehydroascorbate (ASA/DHA) and GSH/GSSG ratios, glutathione reduction potential and freezing tolerance in wheat [33]. Besides their involvement in cold acclimation, ascorbate and glutathione are also involved in vernalization. The flowering time of ASA-deficient *Arabidopsis* mutants was shifted substantially [34]. The overexpression of the first enzyme in glutathione biosynthesis led to earlier flowering and an increased GSSG level even at optimal growth temperature [35]. A similar alteration was only observed in wild-type *Arabidopsis* at 4°C. Thus, it was suggested that an increase in GSSG content or changes in the reduction potential of glutathione partially mimicked seed vernalization treatment [35]. Alterations in the GSSG content may influence flowering time through the OXIDATIVE STRESS2 (OXS2) transcription factor [36].

Based on the cited results it was hypothesized that changes in the redox potential of glutathione may affect freezing tolerance and the initial development of flower primordia in wheat. It could be predicted that the pharmacological modification of the redox state of glutathione and its precursors would modify the thiol-dependent redox potential in winter wheat genotypes even at optimum growth temperature and in spring wheat genotypes only at low temperature, since the latter usually activate the protective mechanisms after stronger environmental effects. This hypothesis was tested by comparing freezing tolerance and the initial development of flower primordia after the pharmacological modification of the glutathione redox state in one winter and one spring wheat genotype. The effect of redox changes on the expression of genes related to freezing tolerance and the initial development of flower primordia was studied.

## Results

### Changes in the amount and redox state of thiols

**Figure 1:**
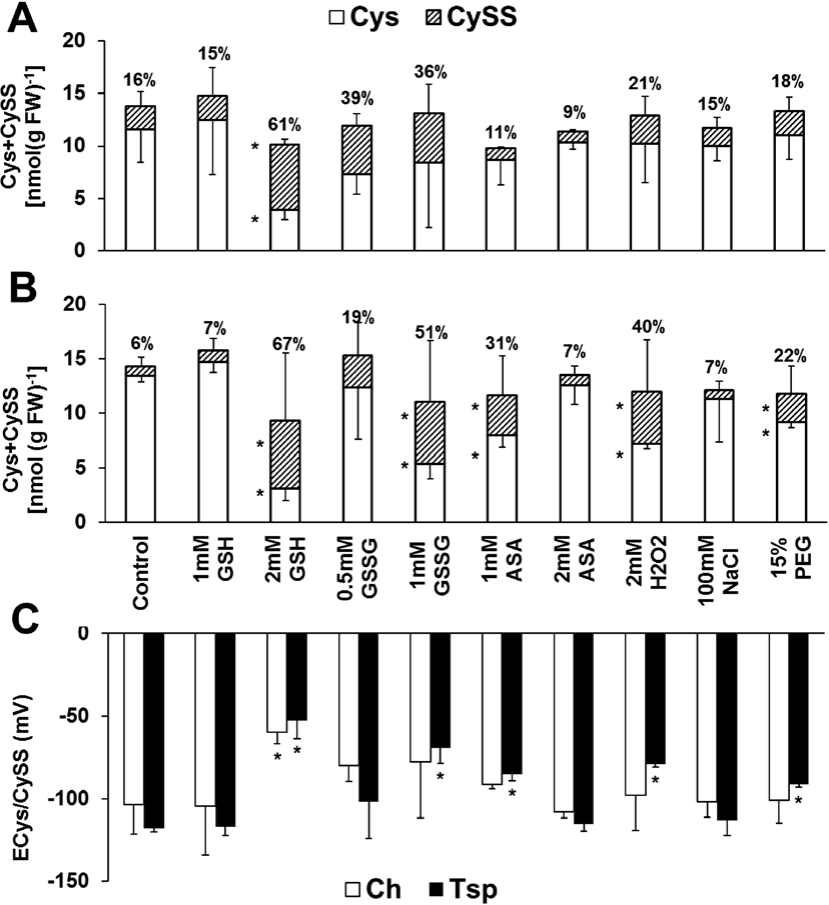
**Pharmacological modification of cysteine content and its reduction potential at low temperature.** The Cys and CySS concentrations of Ch **(A)** and Tsp **(B)** and the reduction potential of both genotypes **(C)** were determined in the crowns of the wheat seedlings. The various compounds were applied to 12-day-old seedlings at 20/17°C for 3 days and subsequently at 5°C for 4 days. The numbers above the columns show the percentage of CySS compared to the total cysteine content. Values indicated by asterisks are significantly different from the corresponding control of each genotype, treated with no chemicals, at the P ≤ 0.05% level.

Twelve-day old seedlings (germination 6 d, growth 6 d) were treated with various reductants (1 and 2 mM GSH and ASA), oxidants (0.5 and 1 mM GSSG, 2 mM H_2_O_2_) and osmotica (15% polyethylene glycol – PEG, 100 mM NaCl) for 3 d at 20/17°C (day/night) as a pre-treatment in order to modify the concentration of the reduced and disulphide forms of thiols and their redox state. The treatments were also continued on the first 4 d of the subsequent cold treatment at 5°C in order to compare the effect of the various compounds at optimal and low growth temperature. The effect of the chemicals on the alteration of the redox environment was monitored by determining the concentration of thiol disulphides and their reduction potential in the crown. The crown plays a special role in cold acclimation and vernalization, since winter wheat genotypes regenerate from this organ after frost damage, and the crown is the place where the very sensitive flower primordia are formed. Treatment with 1 and 2 mM GSH, 1 mM GSSG and 2 mM ASA at 20/17°C decreased the cysteine (Cys) content, and increased the amount of cystine (CySS), the percentage of CySS and the half-cell reduction potential of the cysteine/cystine couple (E_Cys/CySS_) compared with the control in the winter wheat Ch (Additional file 1). In contrast, in the spring wheat Tsp the Cys concentration increased, whereas the content and percentage of CySS and the E_Cys/CySS_ value decreased after the majority of the chemical treatments. However, 1 mM GSH and 2 mM ASA did not affect and 1 mM ASA decreased the Cys content; 1 mM ASA did not change the percentage of CySS and increased the E_Cys/CySS_ value in Tsp. When the temperature was decreased from 20/17°C to 5°C, the Cys content was only decreased and the CySS concentration and the E_Cys/CySS_ value were only increased by 2 mM GSSG compared with the control in Ch (Figure 1). However, at 5°C the Cys content decreased, and the CySS concentration and percentage and the E_Cys/CySS_ value increased after almost all of the treatments compared with the control except after 1 mM GSH, 0.5 mM GSSG, 2 mM ASA and NaCl in Tsp. Among the applied compounds H_2_O_2_ and PEG had significant effects on the amount and redox state of cysteine at both temperatures in Tsp.

**Figure 2:**
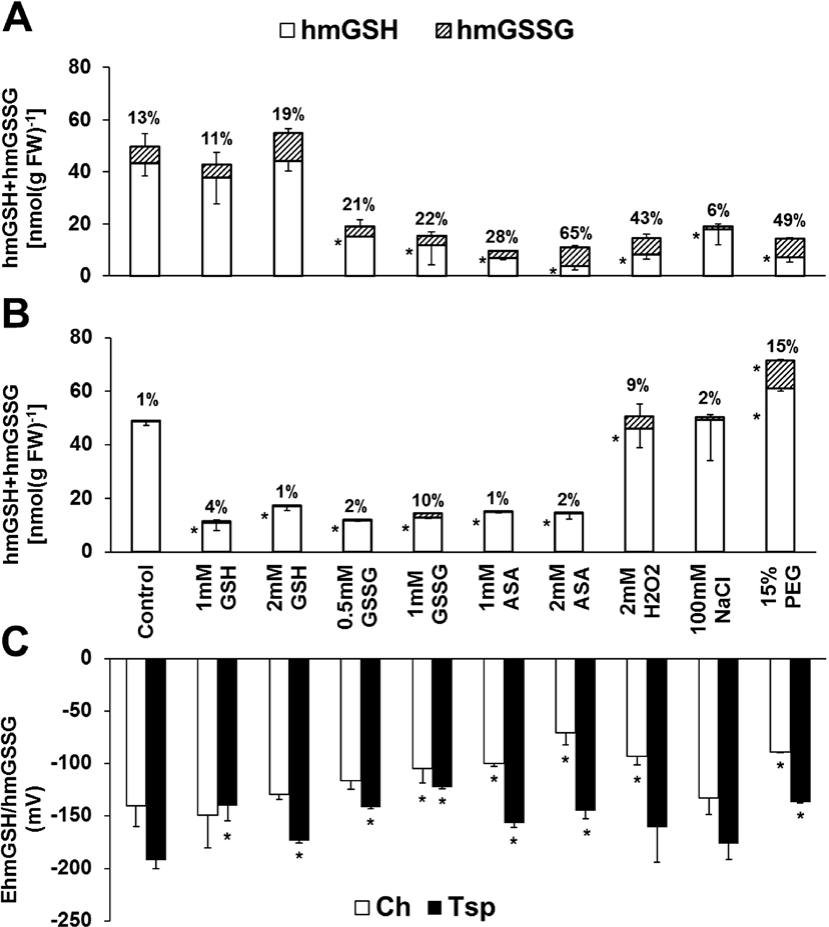
**Pharmacological modification of hydroxymethylglutathione content and its reduction potential at low temperature.** The hmGSH and hmGSSG concentrations of Ch **(A)** and Tsp **(B)** and the reduction potential of both genotypes **(C)** were determined in the crowns of the wheat seedlings. The experimental conditions are described in the legend of Figure 1. The numbers above the columns show the percentage of hmGSSG compared to the total hydroxymethylglutathione content. Values indicated by asterisks are significantly different from the corresponding control of each genotype, treated with no chemicals, at the P ≤ 0.05% level.

Most of the treatments, except for 1mM GSH and 2 mM H_2_O_2_ increased the amount and percentage of hydroxymethylglutathione disulphide (hmGSSG) and the half-cell reduction potential of the hmGSH/hmGSSG couple (E_hmGSH/hmGSSG_) compared with the control at 20/17°C in Ch. The hmGSH content was increased and the E_hmGSH/hmGSSG_ value was decreased by 2 mM ASA, H_2_O_2_, NaCl and PEG in Tsp (Additional file 2). In addition, 1 mM GSH decreased the E_hmGSH/hmGSSG_ value and 2 mM GSH increased it together with the GSSG content in Tsp. At low temperature a great decrease in hmGSH content and an increase in hmGSSG percentage was observed compared with the control except after the addition of both concentrations of GSH in Ch (Figure 2). The E_hmGSH/hmGSSG_ value was increased by 1 mM GSSG, 1 and 2 mM ASA, H_2_O_2_ and PEG in Ch. The hmGSH content decreased and the E_hmGSH/hmGSSG_ value increased compared with the control, except after H_2_O_2_, NaCl and PEG application at 5°C in Tsp.

**Figure 3:**
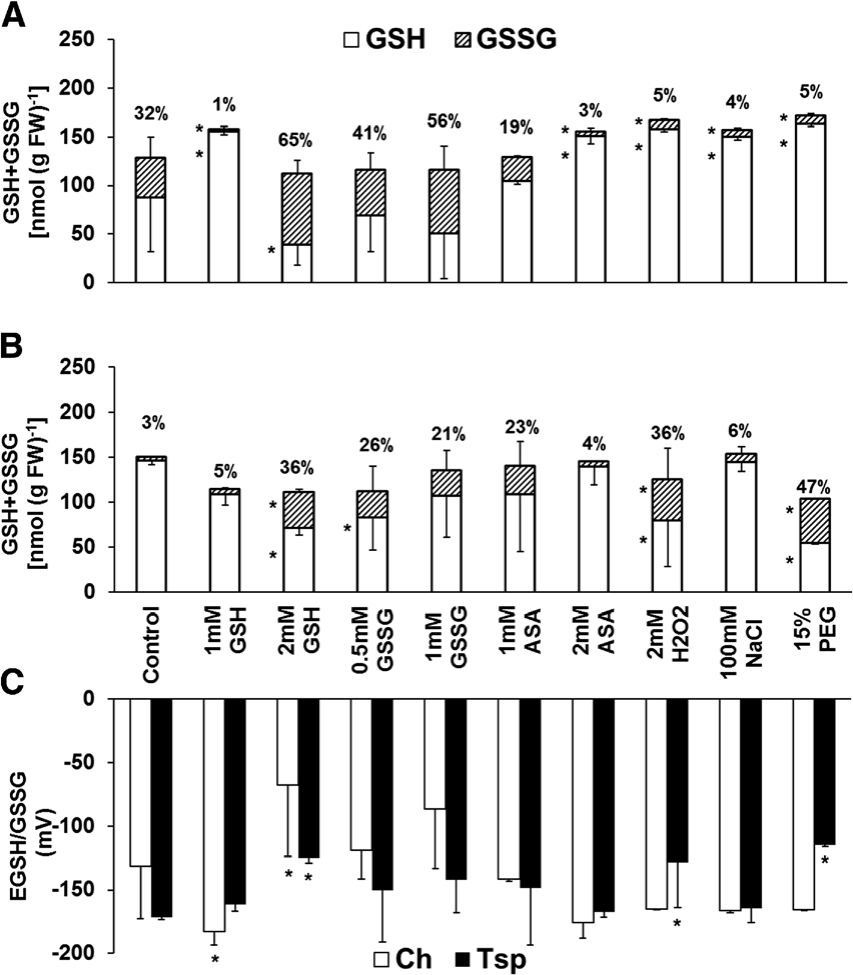
**Pharmacological modification of glutathione content and its reduction potential at low temperature.** The GSH and GSSG concentrations of Ch **(A)** and Tsp **(B)** and the reduction potential of both genotypes **(C)** were determined in the crowns of the wheat seedlings. The experimental conditions are described in the legend of Figure 1. The numbers above the columns show the percentage of GSSG compared to the total glutathione content. Values indicated by asterisks are significantly different from the corresponding control of each genotype, treated with no chemicals, at the P ≤ 0.05% level.

The GSH content was decreased and the GSSG concentrations and the E_GSH/GSSG_ value were increased by 2 mM GSH, 0.5 and 1 mM GSSG, 2 mM ASA and NaCl compared with the control at 20/17°C in Ch (Additional file 3). There was only a slight change, if any in the amount and redox state of glutathione in Tsp. Consequently, there were great differences between the two genotypes for these parameters after treatment with 2 mM GSH, 0.5 and 1 mM GSSG, 2 mM ASA and NaCl. At low temperature the percentage of GSSG was high in control plants, after 2 mM GSH and 1 and 2 mM GSSG and 1 mM ASA treatments, but was lower than in the control following treatment with 1 mM GSH, 2 mM ASA, H_2_O_2_ or osmotica in Ch (Figure 3). The percentage of GSSG was increased by most of the treatments except after 2 mM ASA, the amount of GSSG was increased and the concentration of GSH was decreased by 2 mM GSH, H_2_O_2_ and PEG compared with the control at 5°C in Tsp. The E_GSH/GSSG_ value was decreased by 1 mM GSH and increased by 2 mM GSH in Ch and it was increased by 2 mM GSH, H_2_O_2_ and PEG in Tsp compared with the control.

### Effect of the compounds on fresh weight

**Figure 4:**
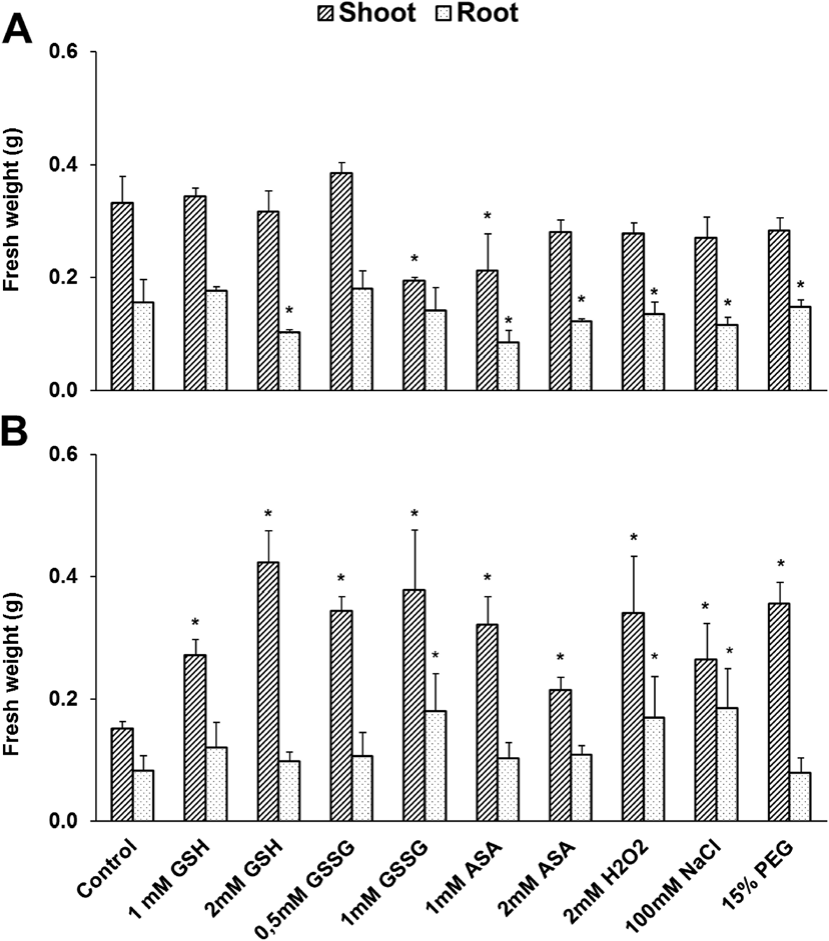
**Effect of redox and osmotic treatments on the fresh weight at low temperature.** The fresh weight of the shoots and roots of Ch **(A)** and Tsp **(B)** is shown. The experimental conditions are described in the legend of Figure 1. Values indicated by asterisks are significantly different from the control, treated with no chemicals, at the P ≤ 0.05% level.

Fresh weight was determined at the same sampling points as the thiol levels after 3 (20/17°C) and 7 days (last 4 d at 5°C) of chemical treatment. Most of the applied compounds had no effect on fresh weight after 3 d at 20/17°C (Additional file 4). The fresh weight of the shoots was not affected (except for the decrease after 1 mM GSSG and 1 mM ASA) and the fresh weight of the roots was reduced (except after 1 mM GSH, 0.5 and 1 mM GSSG) by almost all the treatments compared with the control at 5°C in Ch (Figure 4A). In contrast to Ch, the fresh weight of the shoots was significantly increased by all compounds, whereas the fresh weight of roots was increased by 1 mM GSSG, H_2_O_2_ and NaCl at 5°C in Tsp (Figure 4B).

### Redox regulation of gene expression

**Figure 5:**
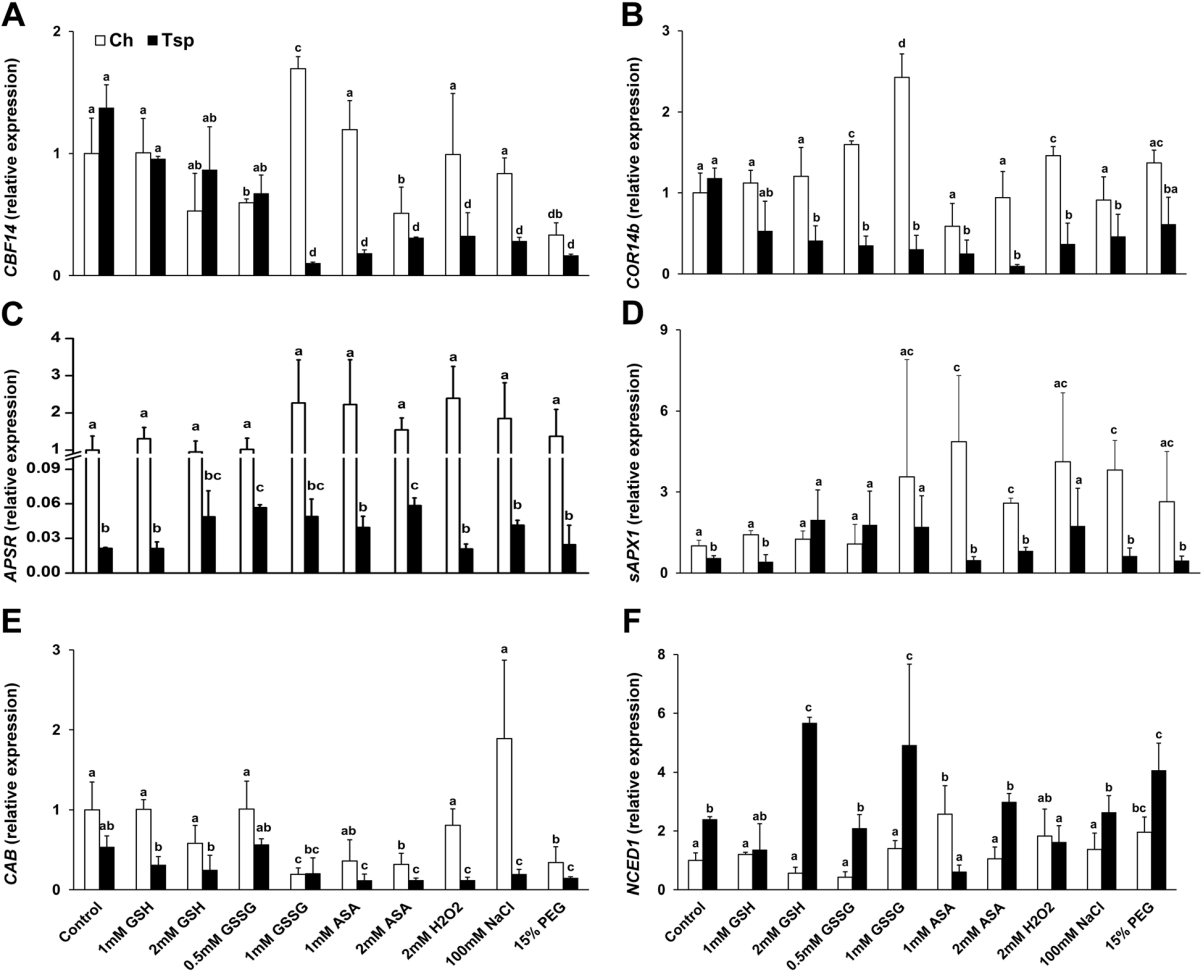
**Effect of redox and osmotic treatments on the expression of genes related to freezing tolerance at low temperature.** The transcription of the genes *CBF14 ***(A)**, *COR14b ***(B) ***APSR ***(C)**, *sAPX1 ***(D)**, *CAB ***(E)** and *NCED1 ***(F)**, related to cold acclimation and antioxidant defence, was investigated in the crown. Gene expression is given as a relative value, based on the values in the control sample of Ch treated with no chemicals. The experimental conditions are described in the legend of Figure 1. The values indicated by different letters are significantly different at p < 0.05 level.

The expression of the genes related to freezing tolerance and the initial development of flower primordia was determined after 7 d treatment with the various compounds (3 d at 20/17°C and subsequently 4 d at 5°C). Figure 5 shows the expression changes observed for the genes involved in the control of freezing tolerance. In Ch the *CBF14* transcript levels exhibited a decrease after treatment with 0.5 mM GSSG, 2 mM ASA and 15% PEG, and an increase after the addition of 1 mM GSSG compared with the control (Figure 5A). In Tsp the *CBF14* expression was strongly reduced except after 1 and 2 mM GSH and 0.5 mM GSSG treatments. Comparing the two genotypes, *CBF14* transcription was lower in Tsp than in Ch after all the treatments, except after both concentrations of GSH, 0.5 mM GSSG and 15% PEG. The expression of *COR14b* was not affected by most of the treatments (except after 0.5 and 1 mM GSSG and H_2_O_2_) in Ch but was decreased by most of them (except after 1 mM GSH and PEG) compared with the control in Tsp (Figure 5B). Two- to four-fold differences were observed between the two genotypes with higher transcript levels in Ch. The transcription of *adenosine-5′-phosphosulphate reductase* (*APSR,* key enzyme of Cys synthesis) was not significantly affected by the treatments in Ch, but was increased by 0.5 mM GSSG and 2 mM ASA in Tsp compared with the control (Figure 5C). The transcript levels of *APSR* were at least 10-fold greater in Ch than in Tsp. The expression of the *stroma ascorbate peroxidase1* (*sAPX1*, degrades H_2_O_2_) gene was increased by 1 and 2 mM ASA and NaCl in Ch, and by 2 mM GSH, 0.5 and 1 mM GSSG and H_2_O_2_ in Tsp compared with the control (Figure 5D). The *sAPX1* transcript levels were at least 2-fold greater in Ch than in Tsp after most treatments, except after 2 mM GSH, 1 and 2 mM GSSG and H_2_O_2_. The expression of the gene encoding a cold-responsive Ca-BINDING protein (CAB) was only reduced by 1 mM GSSG, 2 mM ASA and PEG in Ch; however, in Tsp it was lower after most treatments compared with the control except after both concentrations of GSH and GSSG treatments (Figure 5E). *CAB* expression was 2- to 3-fold greater in Ch than in Tsp except after 1 mM GSSG. To establish whether the effect of the applied chemicals on freezing tolerance was mediated by ABA, the expression of the gene encoding *9-cis-epoxycarotenoid dioxygenase* (NCED1), the regulatory enzyme of ABA synthesis was measured. Its expression was increased by 1 mM ASA and PEG in Ch and by 2 mM GSH, 1 mM GSSG and PEG in Tsp compared with the control (Figure 5F). The transcript level of *NCED1* was greater in Tsp than in Ch after most of the treatments except after 1 mM GSH, 1 mM ASA, H_2_O_2_ and PEG.

**Figure 6:**
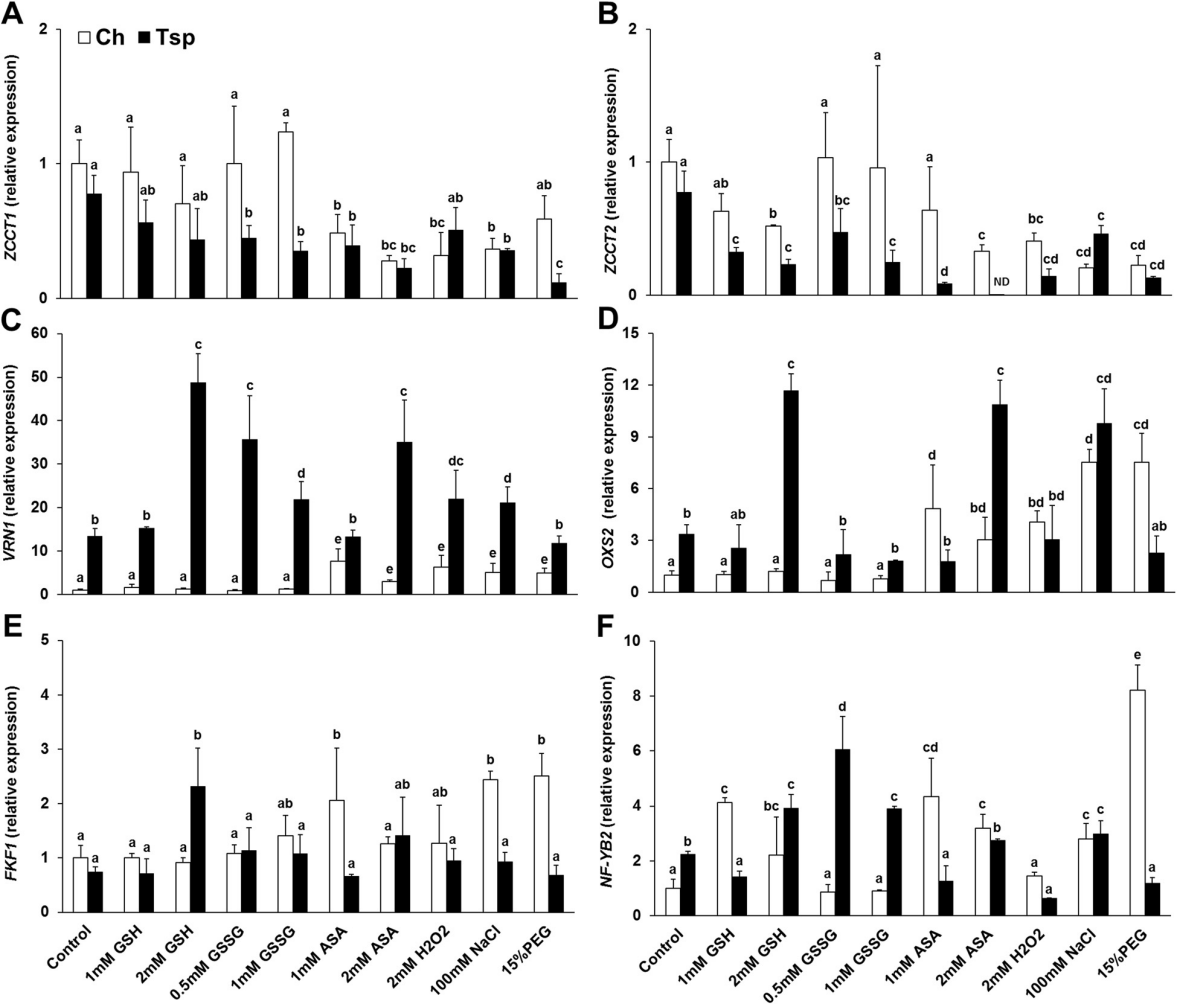
**Effect of redox and osmotic treatments on the expression of genes related to the initial development of flower primordia at low temperature.** The transcription of the genes *ZCCT1 ***(A)**, *ZCCT2 ***(B)**, *VRN1 ***(C)**, *OXS2 ***(D)**, *FKF1 ***(E)** and *NF-YB2 ***(F)**, related to the initial development of flower primordia, was investigated in the crown. Gene expression was given as a relative value, based on the values in the control sample of Ch treated with no chemicals. The experimental conditions are described in the legend of Figure 1. The values indicated by different letters are significantly different at p < 0.05 level. ND: not detectable.

Among the genes controlling the initiation of the flower primordia, the expression of the flowering repressor *ZCCT1* was not affected by 1 and 2 mM GSH, GSSG and 15% PEG, but was reduced by the other treatments in Ch, whereas it was reduced by most of the treatments in Tsp except after 1 and 2 mM GSH and H_2_O_2_ compared with the control (Figure 6A). A significant difference between the two genotypes in *ZCCT1* transcription was only observed after the addition of 0.5 and 1 mM GSSG and 15% PEG. The transcript level of the *ZCCT2* gene generally decreased in both genotypes compared with the control except after 1 mM GSH, 0.5 and 1 mM GSSG and 1 mM ASA in Ch, and this change was much greater in Tsp (Figure 6B). In contrast to *ZCCT1*, the expression of *ZCCT2* differed greatly between Ch and Tsp after treatment with redox agents and osmotica. It was at least 2-fold greater in Ch than in Tsp except after NaCl and PEG addition. The transcription of *VRN1* was not affected by either concentration of GSH or by GSSG, but was increased 2- to 4-fold by the other treatments in Ch compared with the control (Figure 6C). The expression of *VRN1* was induced by most of the compounds except after 1 mM GSH, 1 mM ASA and PEG in Tsp. The transcript levels of *VRN1* were 2- to 10-fold greater in Tsp than in Ch. The transcripts of *VRN3*, which is a positive regulator of flowering were not present at a detectable level in the crowns. The expression of *OXIDATIVE STRESS2* (*OXS2*), which controls stress-induced flowering was greatly induced by 1 and 2 mM ASA, NaCl, H_2_O_2_ and PEG in Ch and by 2 mM GSH, 2 mM ASA and NaCl in Tsp compared with the control (Figure 6D). The transcript level of *OXS2* was higher in Tsp than in Ch after the addition of 2 mM GSH, 0.5 and 1 mM GSSG and 2 mM ASA. The transcription of *FLAVIN-BINDING KELCH-REPEAT-BOX1* gene (*FKF1*), another regulator of flowering time was induced by 1 mM ASA, NaCl and PEG in Ch and by 2 mM GSH in Tsp compared with the control (Figure 6E). The expression of *FKF1* was greater after 1 mM ASA, NaCl and PEG addition in Ch and after the application of 2 mM GSH in Tsp compared to the other genotype. The transcript level of the stress-responsive *NF-YB2* was increased by most treatments, except after 0.5 and 1 mM GSSG and H_2_O_2_ compared with the control in Ch (Figure 6F). The expression of *NF-YB2* was elevated by 2 mM GSH, 0.5 and 1 mM GSSG and NaCl and decreased after 1 mM GSH, 1 mM ASA, H_2_O_2_ and PEG treatments in Tsp. Greater transcript levels were detected after the addition of 1 mM GSH, 1 mM ASA and PEG in Ch and after treatment with 0.5 and 1 mM GSSG in Tsp compared with the other genotype.

### Redox control of freezing tolerance

**Figure 7:**
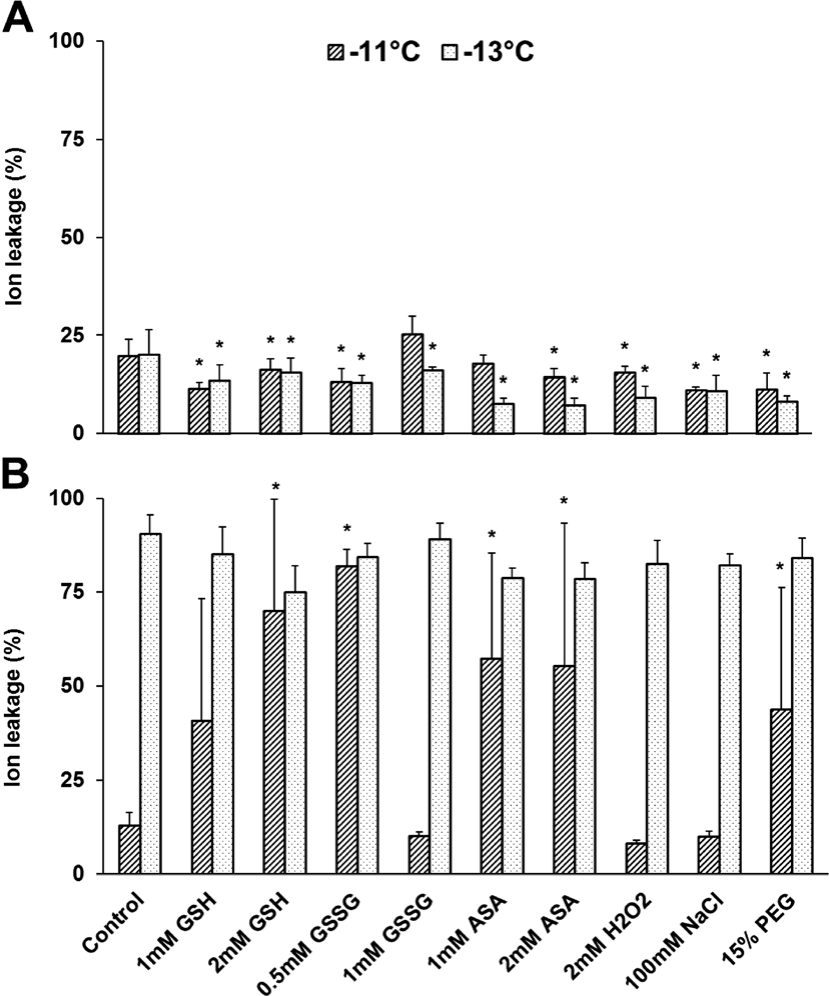
**Effect of redox and osmotic treatments on freezing tolerance.** Electrolyte leakage was measured in leaf segments of Ch **(A)** and Tsp **(B)** after 3 weeks of cold hardening at -11°C and -13°C. Various compounds were applied to 12-day-old seedlings of Ch and Tsp at 20/17°C for 3 days and subsequently at 5°C during the first 4 days of the 3-week cold hardening period. High values of electrolyte leakage indicate severe damage to the cell membranes and high freezing sensitivity. Values indicated by asterisks are significantly different from the control, treated with no chemicals, at the P ≤ 0.05% level.

Freezing tolerance was tested by measuring the electrolyte leakage as an indicator of membrane damage after freezing of the leaf segments of the cold-hardened plants (3 weeks, 5°C) at different temperatures. The temperatures for freezing and the 2°C difference between them were based on previous results [37]. The compounds applied improved freezing tolerance as shown by the decrease in electrolyte leakage at both temperatures compared with the control except for 1 mM GSSG and 1 mM ASA at -11°C in Ch (Figure 7). They reduced the tolerance as indicated by the increase in electrolyte leakage except after 1 mM GSH, 1 mM GSSG, H_2_O_2_ and NaCl treatments at 11°C in Tsp. The damage suffered by the freezing-sensitive spring wheat Tsp was lethal even without chemical treatment at -13°C. The test was also carried out at -15°C, but the electrolyte leakage was almost 100% even in the freezing-tolerant genotype after all treatments indicating the high damage of cell membranes (data not shown).

### Effect of redox treatments on the initial development of flower primordia and H_2_O_2_ accumulation in the shoot apices

**Figure 8:**
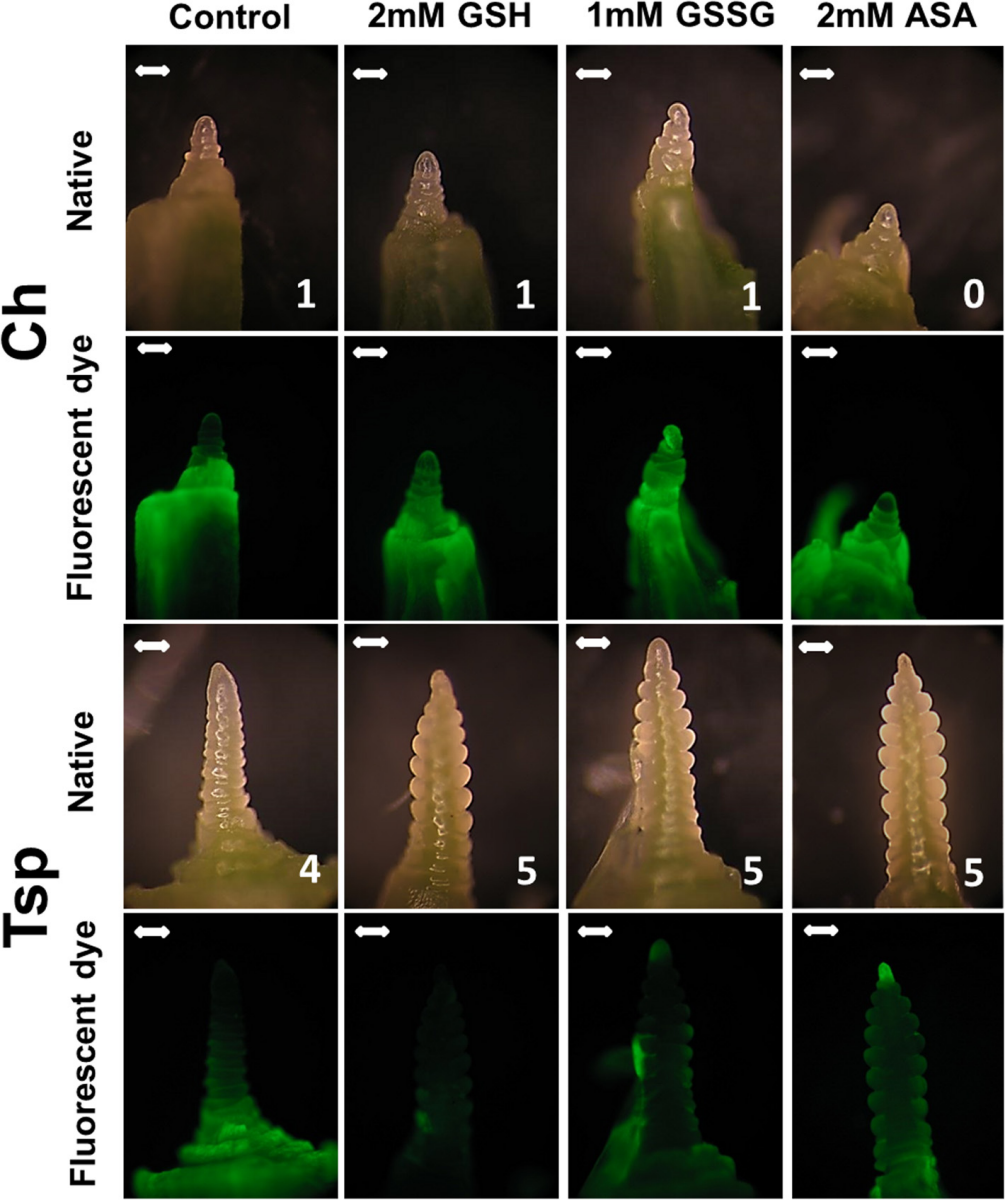
**Effect of redox and osmotic treatments on shoot apex morphology and peroxide content.** Apices were isolated at the end of the 3-week recovery phase to check the effect of the treatments on the vegetative/generative transition (first and third rows). The peroxide content was detected with the green fluorescent dye H_2_DCFDA (second and fourth rows). The various compounds were applied to 12-day-old seedlings of Ch and Tsp at 20/17°C for 3 days and subsequently at 5°C during the first 4 days of the 3-week cold hardening period, which was followed by a 3-week recovery period at 20/17°C. Photos of the apices after the other treatments can be seen in Additional file 5. The numbers on the native photos indicate the developmental stage of the flower primordia according to the following scale: 0 – vegetative apex, 1 – start of apex elongation, 2 – elongation with single ridge, 3 – double ridge indicating the vegetative/generative transition, 4 – enlargement of spikelet primordia, 5 – empty glume primordia [51]. The bars indicate 200 μm.

The initial development of flower primordia was monitored by investigating shoot apex morphology at the end of the 3-week recovery period. This process was not affected in Ch and was accelerated by most of the treatments in Tsp (Figure 8, Additional file 5). The shoot apices of Ch were in developmental stages 0–2 (before the generative transition) both with and without chemical treatment. However, in Tsp the control apices were in stage 4, in which the spikelet primordia enlarge, whereas after the addition of the various compounds the apices were in stages 5–6, which are called the ‘empty and lemma glume primordia’ stages. The isolated apices were stained with the green fluorescent dye H_2_DCFDA in order to investigate the peroxide concentration at the end of the 3-week recovery period. This was slightly increased by both concentrations of ASA and GSH and by 1 mM GSSG in Ch, and was decreased by most of the chemicals except after 2 mM ASA and NaCl in Tsp (Figure 8, Additional file 5).

## Discussion

### Modification of the redox state of thiols

It was shown that the redox state of the thiols was modified by the addition of reductants, oxidants and osmotica to the nutrient solution in hydroponically grown wheat seedlings. The redox state of glutathione was affected not only by GSH and GSSG, but also by ASA, H_2_O_2_, NaCl and PEG, indicating that this modification was not a simple feed-back control of its synthesis or reduction by the substrate, but part of a more general redox control process. ASA and H_2_O_2_ may affect the redox state of glutathione through the ascorbate-glutathione cycle, whereas NaCl and PEG may influence it through the osmotic stress-induced accumulation of H_2_O_2_. Changes in the GSSG content and E_GSH/GSSG_ value, which were closely correlated with each other (Additional file 6) were only observed at optimal growth temperature in the freezing-tolerant Ch but not in Tsp after treatment with the various compounds, leading to great differences in these parameters between the treated seedlings of the two genotypes. At 20/17°C the E_GSH/GSSG_ value was generally increased significantly by the treatments in Ch compared to the control, whereas there was no significant change in Tsp. However, if the chemical treatments were combined with cold (5°C), the E_GSH/GSSG_ value exhibited a similar general change in Tsp like the one observed for Ch at 20/17°C, whereas it was partly restored to the value detected before the cold treatment in Ch. These differences between the two genotypes may be due to the different levels of antioxidants before the treatments, as shown by the higher GSSG content and E_GSH/GSSG_ value in Ch compared to Tsp, and result in the different expression of genes related to freezing tolerance and the initial development of flower primordia in the two genotypes. This is supported by the fact that a change (20 mV) in the E_GSH/GSSG_ value similar to that observed for wheat in the present study dramatically decreased the seed viability of four plant species [38].

Besides gluthathione, the other two thiols, cysteine and hydroxymethylglutathione may also modify the cellular redox environment, and consequently the structure and activity of redox-responsive molecules [39]. However, changes in the redox state of glutathione may have the greatest effect on the redox environment, since its concentration was 3- to 4-fold greater than that of hydroxymethylgluthione and 10-fold greater than that of cysteine. The importance of the maintenance of the appropriate glutathione redox state is also indicated by the contrasting effect of 1 mM and 2 mM GSH on the redox state of cysteine and glutathione. This difference may be explained by the GSH sensitivity of the key enzyme of cysteine synthesis, *adenosine-5'-phosphosulfate reductase*[40]. Accordingly, we assume that it is not affected by the 1 mM GSH concentration, but may be severely inhibited by the 2 mM GSH concentration. Consequently, the amount of Cys which is the precursor of GSH, as well as the GSH concentration will be reduced by 2 mM GSH. The marked increase in CySS and GSSG may be explained by the severe inhibition of cysteine reductase and glutathione reductase by 2 mM GSH [41].

It should be mentioned that at 20/17°C the various compounds added only induced an increase in the concentration of the disulphide forms and half-cell reduction potential of glutathione and the two other thiols in Ch. At 5°C, however, the redox state of both GSH and cysteine was similar in the two wheat genotypes, but in Tsp the percentage of hmGSSG was only 1–2%, and the total level (reduced + disulphide forms) was decreased to 20–30% of the control value after the majority of the treatments. By contrast, the ratio of hmGSSG was increased (to 21–65%) by nearly all treatments at 5°C in Ch. Based on this difference between the winter and spring wheat genotypes, the hmGSH/hmGSSG couple may have a special role in the regulation of the redox-responsive molecules involved in cold acclimation and the initial development of flower primordia in Poaceae*,* where hmGSH is a homologue of GSH (the cysteine is replaced by a serine).

The influence of cold on redox changes described earlier [33] was intensified when combined with various chemical treatments in the present study, both in Ch and Tsp. Both the combined application of cold and various redox agents and cold treatment alone had a greater effect on the redox system in Ch than in Tsp, and there was a strong correlation between freezing tolerance and redox changes [33]. The effect of exogenous GSH on tolerance to low temperature was also shown in tobacco [42]. In addition, PEG-induced osmotic stress resulted in greater changes in the amount and redox state of glutathione in a tolerant wheat genotype than in a sensitive one [43].

The redox state can be modified not only by various pharmacological compounds [44], but also by the overexpression or inhibition of the related enzymes. Thus, the increased expression of a gene encoding an enzyme with both glutathione S-transferase and glutathione reductase activities affected the amount of glutathione and its redox state in tobacco [42]. Changes in the activity of these and other enzymes may lead to the oxidation of GSH and indirectly to that of other compounds involved in the ascorbate-glutathione cycle, and to changes in the cellular redox potential [28]. Similar redox changes were described in mutants deficient in ascorbate and glutathione or in the enzymes involved in the reduction of their oxidised forms, leading to an increase in the cytosolic redox potential compared to wild-type plants [28]. Similarly to the pharmacological modification of the redox state, the use of hypomorphic mutants or RNAi transgenic lines would also allow the cellular redox environment to be modified gradually, thus facilitating the study and promoting the understanding of its regulatory role.

Although monitoring the endogenous redox changes induced by various environmental effects makes it possible to clarify their role in growth, development and the stress response, the pharmacological modification of the levels of various redox components is an important tool to obtain additional information about their participation in these processes, as shown in the case of chilling in maize [45].

### Redox control of freezing tolerance

The importance of endogenous redox changes during cold acclimation and their correlation with freezing tolerance was shown in wheat seedlings [33]. The exogenous application of redox compounds and osmotica induced a great increase in oxidized thiols and simultaneously increased freezing tolerance in the winter wheat genotype Ch, but not in the spring genotype Tsp. Comparing the effect of the various compounds tested, it can be concluded that, rather than having specific effects, the individual compounds have a similar influence on the ascorbate-glutathione cycle and on the redox potential of the GSH/GSSG couple, resulting in an improvement in freezing tolerance. The increase in the amount of GSSG could be important in this process, since the higher tolerance of transgenic tobacco seedlings to salt and chilling stress was also related to the elevated GSSG concentrations [42]. Changes in the amount and ratio of GSH and GSSG may influence the metabolism through the thiol/disulphide conversion or the (de)glutathionylation of proteins, which modifies their activity. Changes in the Cys/CySS and hmGSH/hmGSSG ratios may have a similar effect on proteins and subsequently on freezing tolerance as shown by the different effects of 0.5 mM and 1 mM GSSG on the ratio of disulphide forms and subsequently on freezing tolerance in Tsp. The redox potential of glutathione showed a moderate correlation with freezing tolerance (r^2^: 0.64) in Ch (winter wheat), whereas there was no correlation in Tsp (spring wheat) (r^2^: 0.08), indicating that the redox changes induced by the various treatments tested only improved freezing tolerance in the winter genotype.

**Figure 9:**
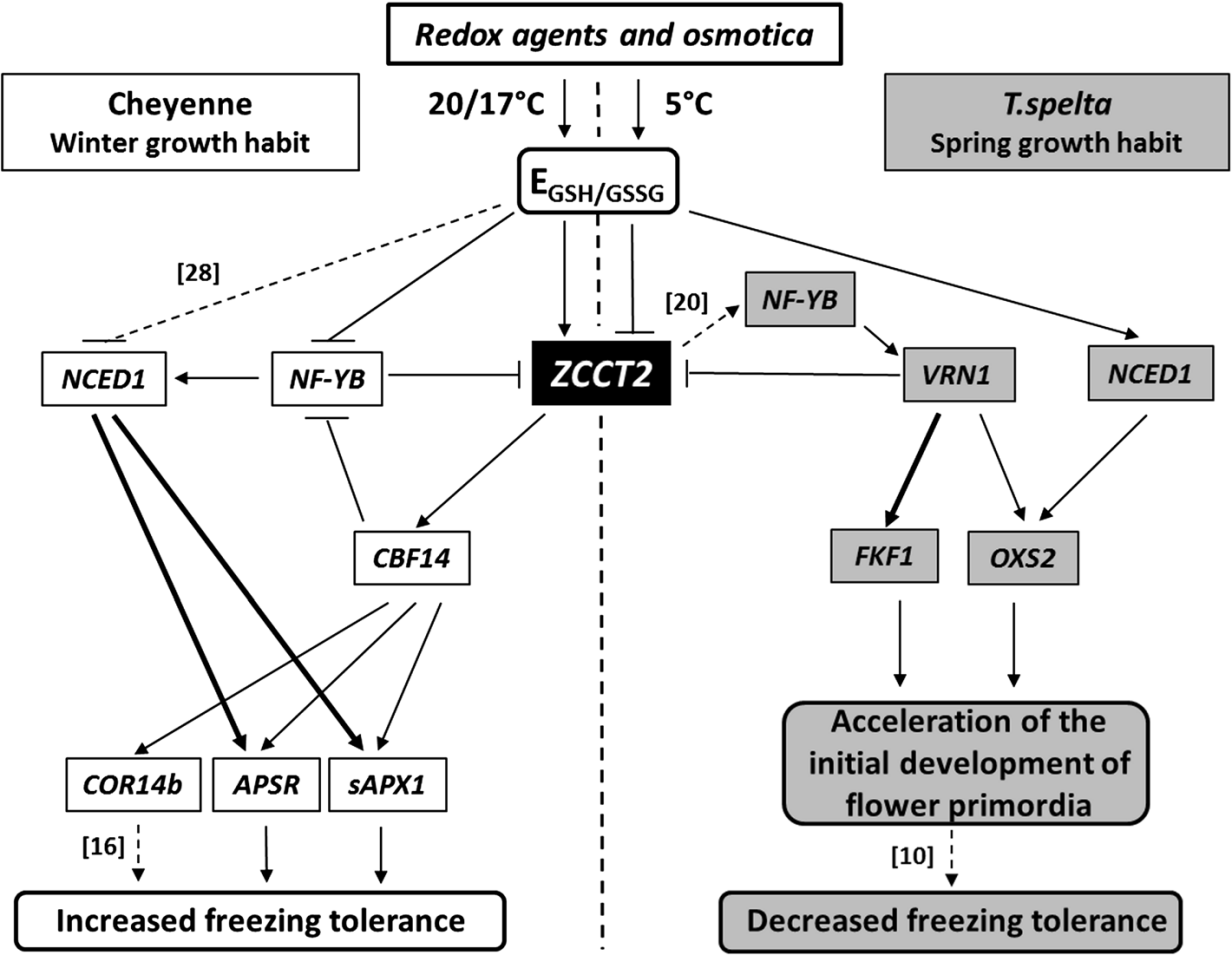
**Central role of *ZCCT2 *in the redox control of freezing tolerance and the initial development of flower primordia.** The redox compounds and osmotica induced different timing and level of changes in the E_GSH/GSSG_ value and subsequently in *ZCCT2* expression in Cheyenne (Ch, left side) and *T. spelta* (Tsp, right side). The chemical treatments induced a great increase in E_GSH/GSSG_ value after 3 d at 20/17°C in Ch and during the subsequent cultivation for 4 d at 5°C in Tsp. Higher *ZCCT2* transcript levels in Ch were generally associated with greater expression of genes (*CBF14, APSR, sAPX1*) that contribute to increased freezing tolerance. *NF-YB* may affect *ZCCT2* in a regulatory loop, which also influences the ABA-dependent freezing tolerance through *NCED1*. The lower level of *ZCCT2* in Tsp was associated with induction of the regulators activating the initial development of flower primordia (*OXS2, FKF1, VRN1*). Based on the correlation analysis, ABA (*NCED1*) may also be involved in the redox-dependent regulation of the initial development of flower primordia. Continuous lines indicate the proposed relationship between two parameters based on the correlation analysis (thin line: moderate correlation, thick line: close correlation) and broken lines refer to data from the literature indicated by the corresponding numbers.

A model was created to explain the different responses of the two genotypes to various redox agents and osmotica, based on differences in E_GSH/GSSG_ values, gene expression, freezing tolerance and the initial development of flower primordia, and on correlations between these parameters (Figure 9). Based on correlation analysis (Additional file 6), the different effects of the chemicals on the E_GSH/GSSG_ values in the two genotypes (induction of an increase already at 20/17°C in Ch and only at 5°C in Tsp) may contribute to the *ZCCT2* transcript level’s being, on the average, 2-fold higher in Ch than in Tsp at 5°C. This difference in *ZCCT2* transcript levels may be responsible for its different effect on freezing tolerance and the initial development of the flower primordia in the two genotypes. Interestingly, such a difference in *ZCCT1* expression between the two genotypes was only observed after few treatments. The expression of *ZCCT2* and *ZCCT1* exhibited similar correlations with the transcript levels of the other genes. The redox sensitivity of *ZCCT2* was also shown in another wheat genotype, Chinese Spring, in which a short treatment (3 h) with H_2_O_2_ resulted in a 2- to 3-fold increase in its expression (G. Kocsy, unpublished results). According to a recent paper *ZCCT1* and *ZCCT2* expression is inhibited by *VRN1*[13]. The negative correlation found between the expression levels of these genes in both genotypes was close in Ch and moderate in Tsp. The great increase in *VRN1* transcript level generally observed was associated with a great reduction in *ZCCT2* transcript level after the majority of chemical treatments in Tsp, whereas the decrease in *ZCCT2* transcription was only moderate for the winter wheat Ch. Thus, the higher expression level of *ZCCT2* in Ch is inferred to have been sufficient to keep the plants in the vegetative developmental phase. Correlation analysis showed that the greater transcript level of *ZCCT2* was also associated with a higher expression of *CBF14* and its target genes in Ch compared to Tsp (Additional file 6). Although the expression of *CBF14* was only higher than the control after 4 d treatment with GSSG at 5°C, differences were found for *COR14b* and s*APX1* after several treatments. Interestingly, although GSSG increased the transcription of these genes, GSH did not, an observation which is consistent with the results obtained in tobacco, where a relationship between GSSG content and stress tolerance was found [42]. Due to interactions between the *NF-Y* and *ZCCT2* regulatory proteins [20], *NF-Y* may also be involved in the control of cold-responsive genes through *CBF14*. The negative correlation between *CBF14* and *NF-YB* (Additional file 6) suggests that *CBF14* may be associated with the inhibition of *NF-YB*, which in turn may have a similar effect on *ZCCT2*, forming a feed-back regulatory loop between flowering time regulation and the cold response, as suggested for *Arabidopsis,* with the involvement of the *CBF1*, *SOC1* and *FLC* genes (note that *ZCCT2* has a function similar to that of FLC) [46]. This whole loop is controlled by the E_GSH/GSSG_, which may have a positive effect on *ZCCT2* and a negative influence on *NF-YB* at 5°C in Ch based on our correlation analysis (Additional file 6). The latter may form a link between the ABA-independent and ABA-dependent regulation of cold-responsive genes by controlling *NCED1*, which encodes a key enzyme of ABA synthesis. This hypothesis is in agreement with previous data [22]. Earlier experiments showed that redox changes may affect ABA signalling directly, independently of *ZCCT2* and *NF-Y*[28]. Although in the present experiment the expression of *NCED1* was not correlated with the E_GSH/GSSG_ value, it was in close correlation (r^2^: 0.72 and 0.82) with the transcription of the *APSR* and s*APX1* genes*.* The very close correlation (r^2^: 0.94) observed in Ch between the *APSR* and *sAPX1* transcript levels can be explained by their coordinated regulation through the ascorbate-glutathione cycle. A correlation between redox changes and freezing tolerance-related genes was also shown in Tsp (Additional file 6). There was a very close positive correlation between *ZCCT2* and both *CBF14* and *COR14b*, and a close correlation between *ZCCT2* and *CAB* expression. *CAB* was also closely correlated with *CBF14, COR14b* and *APSR*. However, these genes had low expression in Tsp, which may be explained by the low *ZCCT2* transcript level, which resulted in freezing sensitivity. The relationship between freezing tolerance and differences in the gene expression profile was also shown by the comparison of substitution lines of Tsp and Ch involving chromosome 5A (on which major genes regulating cold acclimation and vernalization are localized) [47]. In a macroarray experiment, about 100 genes were only affected by the 5A chromosome of Tsp and about 150 only by that of Ch. There was a difference in the transcriptome of the two genotypes even before cold treatment. An even larger difference can be assumed between Tsp and Ch in the present experiment, since differences between the two genomes are not restricted to chromosomes 5A, like in the case of the substitution lines. Thus, different gene sets appear to be the target of the redox changes in the two genotypes. Correspondingly, genes related to cold acclimation were expressed to a much greater extent in the freezing-tolerant Ch than in the freezing-sensitive Tsp after the various treatments tested.

### Redox control of the initial development of flower primordia

In contrast to the improvement of freezing tolerance in Ch, a different adaptive strategy was observed in the spring genotype Tsp after the various treatments, involving an accelerated growth of the shoots and roots and a quicker initial development of the flower primordia. The changes observed after the combined application of cold and the various compounds were accompanied by increased GSSG content, which was also involved in the initiation of flowering in *Arabidopsis*[35]. The importance of the fine regulation of GSSG content is also indicated by the stronger effect of its higher concentration on the initial development of flower primordia in Tsp in the present experiment. The redox changes depending on the redox state of glutathione may be important developmental signals affecting the whole metabolism and, consequently, the growth and development of plants. As in wheat, the involvement of ASA in controlling the initial development of flower primordia was also shown in *Arabidopsis*, where flowering was delayed in ASA-deficient mutants under long-day conditions [48]. Whereas in an earlier study the developmental stage of the flower primordia did not correlate with the endogenous level of various antioxidants during the 3-week cold hardening [33], the exogenous application of redox agents accelerated the initial development of flower primordia during the recovery period after growth at low temperature. This contradiction can be explained by the different redox processes occurring during cold treatment and the subsequent recovery, or by the more drastic effect of exogenous redox compounds. The concentrations and oxidation levels of ascorbate and glutathione may affect the flowering time *via* the control of H_2_O_2_ levels through the ascorbate-glutathione cycle. This assumption is confirmed by the present findings, since the effect of exogenous H_2_O_2_ on the initial development of flower primordia was similar to that of GSH and ASA. In addition, a correlation was found between ascorbate peroxidase activity, H_2_O_2_ level and flowering time when an ascorbate peroxidase-deficient mutant was compared to wild-type and overexpressing *Arabidopsis* plants [49]. The mutants, which had the highest H_2_O_2_ content, flowered first and the transgenic plants with the lowest H_2_O_2_ content last. Osmotica may also induce H_2_O_2_ accumulation and subsequently to stress-induced early flowering [48]. The importance of H_2_O_2_ in the control of flowering at the gene expression level was shown by transcriptome analysis in *Arabidopsis*, where H_2_O_2_ increased the expression of a CONSTANS-LIKE protein [36]. The genetic basis of stress-induced early flowering was recently described in plants [36] and the results were used to elaborate a model for the redox regulation of flowering [23].

Based on the present experiment, GSH-dependent redox changes inhibit *ZCCT2* transcription to a greater extent in Tsp than in Ch (Figure 6B). From the negative correlation between *ZCCT2* and *VRN1* transcript levels in Tsp, it can be supposed that the decrease in *ZCCT2* expression may be associated with the increased expression of *VRN1* in the present experiment. The repression of *ZCCT2* (present in the *VRN2* locus) by *VRN1* was reported in a recent study [13]. *ZCCT2* may control *VRN1* transcript levels through its interaction with *NF-YB* in a regulatory loop, in which *NF-YB* may have a positive effect on *VRN1* expression [20] (Additional file 6). As a result of this regulation possibility, *VRN1* expression was much greater in Tsp after the majority of the treatments compared to the control. *VRN1* might have a positive effect on *OXS2* and *FKF1*, which are positive regulators of flowering. According to our hypothesis this led to an accelerated initial development of shoot apices, shown by the more developed flower primordia of seedlings treated with redox compounds and osmotica compared with the control. Although correlation analysis did not reveal any relationship between the expression of *ZCCT2, OXS2* and *FKF1* in wheat (Additional file 6), *ZCCT2* may activate *OXS2* and *FKF1* through *NF-YB* and *VRN1*. Interestingly, the effect of redox changes (E_GSH/GSSG_) on *OXS2* expression may be mediated by ABA based on correlations between E_GSH/GSSG_ value and *NCED1* transcript levels (Additional file 6). This assumption is supported by the results obtained in *Arabidopsis*, where ABA and *OXS2* were found to have an effect on drought-induced early flowering under long-day growth conditions [49]. Besides having a stimulating effect on the initial development of flower primordia, the increased *VRN1* expression in Tsp may also be responsible for the decrease in freezing tolerance, because of the inhibition of cold-responsive genes [16,17]. The coordinated regulation of flowering and tolerance to low temperature was also described in *Arabidopsis*[46]. The redox control of the initial development of flower primordia was shown not only in Tsp but also in Ch, where the low expression of *OXS2* and *FKF1* (which are closely correlated with each other) may be associated with the higher *ZCCT2* transcript level, as indicated by the negative correlation between *ZCCT2* and the other two genes (Additional file 6). Consequently, the initial development of flower primordia was delayed. The effect of ABA on flowering was also indicated by the close correlations between *NCED1*, *OXS2* and *VRN1* in Ch.

## Conclusions

The application of redox-altering compounds (reductants, oxidants and osmotica) differentially affected the GSSG content and the E_GSH/GSSG_ values, and consequently the expression of the flowering repressor *ZCCT2*, in the two genotypes. The much greater expression of *ZCCT2* in Ch compared with Tsp after the various treatments was associated with the much lower expression of *VRN1*, the major regulator of the initial development of flower primordia, and with greater expression of genes increasing freezing tolerance. However, the much smaller *ZCCT2* transcription (due to its strong repression by the various compounds tested) in Tsp compared to Ch was associated with much greater *VRN1* expression and much lower transcript levels of the genes related to freezing tolerance. Based on the correlation between the expression of genes related to the initial development of flower primordia and cold acclimation improving freezing tolerance, a model was constructed to illustrate the coordinated control of the two processes. The effect of the various redox-altering compounds is mediated by alterations in GSSG concentrations and the E_GSH/GSSG_ value in the proposed model, in which *ZCCT2* has a central regulatory role.

## Methods

### Plant material and treatments

A freezing-sensitive, spring habit *Triticum aestivum* ssp. *spelta* (Tsp) accession and the freezing-tolerant, winter habit *Triticum ae.* ssp. *aestivum* cv. *Cheyenne* (Ch) wheat cultivar were studied. Following germination in Petri dishes (1 d 25°C, 3 d 5°C, 2 d 25°C), seedlings were grown on half-strength modified Hoagland solution with a photoperiod of 16 h, at 260 μmol m^-2^ s^-1^, 20/17°C and 70/75% RH in a growth chamber (Conviron PGV-15; Controlled Env., Ltd., Winnipeg, Canada) [32]. Twenty seedlings were cultivated on 500 ml nutrient solution in plastic pots. The solution was changed every week and at the beginning and end of the chemical treatments. After 6 days of growth, various reductants (1 and 2 mM GSH and ASA), oxidants (0.5 and 1 mM GSSG, 2 mM H_2_O_2_) or osmotica (15% PEG, 100 mM NaCl) were added to the nutrient solution as a pre-treatment, in order to observe their influence on the initial development of flower primordia and cold acclimation. GSH, GSSG, ASA and H_2_O_2_ were chosen due to their involvement in the ascorbate-glutathione cycle, to see what changes they induced in the thiol content and redox potential and how these alterations influenced the other parameters investigated, whereas NaCl and PEG were included to determine the effect of the oxidative stress induced by osmotica. The concentrations of the various compounds were determined in preliminary experiments using a dilution series. To compare their effect on the redox environment at temperatures of 20/17°C and 5°C, they were also added to the nutrient solution during the first four days of cold treatment. The 3-week cold hardening was followed by 3 weeks of recovery at 20/17°C. Samples were collected for biochemical analysis and the fresh weight of shoots and roots was measured after 3 (Additional file 4) and 7 days (Figure 4) of treatment with the various compounds. There were 3 independent experiments each with 3 parallel samples.

### Determination of freezing tolerance

Freezing tolerance was estimated at the end of the 3-week cold hardening period by freezing 1 cm leaf segments (covered with aluminium foil and placed in sand in glass tubes) at -11, -13 or -15°C for 1 h. The temperature was decreased to freezing temperatures gradually (2°C for 6 h, -2°C for 15 h, then 2°C decrease every 2 h). The leaf segments were kept at 2°C for 2 h after freezing, then placed in vials containing 10 ml ultrapure water (Milli-Q 50 water purification system) and shaken overnight at room temperature. Membrane injury was determined by measuring the electrolyte leakage with a conductometer, then all the samples were boiled to destroy the cell membranes and the conductivity was determined again. Relative electrolyte leakage was characterised as the ratio of the first and the second values [50]. High values of electrolyte leakage indicate severe damage to the cell membranes and high freezing sensitivity. The data are shown in Figure 7.

### Determination of thiols

The plant material was ground with liquid nitrogen in a mortar, after which 1 ml of 0.1 M HCl was added to 200 mg plant sample. Total thiol content was determined after reduction with dithiothreitol and derivatisation with monobromobimane [32]. For the detection of oxidised thiols, the reduced thiols were blocked with N-ethylmaleimide, and next the excess of N-ethylmaleimide was removed with toluene [31]. Oxidised thiols were reduced and derivatised as described for total thiols. The samples were analysed after the separation of cysteine, γ-glutamylcysteine (γEC), hydroxymethylglutathione (hmGSH, a homologue of GSH in Poaceae) and GSH by reverse-phase HPLC (Waters, Milford, MA, USA) using a W474 scanning fluorescence detector (Waters). The amount of reduced thiols was calculated as the difference between the amounts of total and oxidised thiols. The half-cell reduction potential of the thiol redox couples was calculated using the Nernst equation [30]. Data referring to Cys, hmGSH and GSH after 3 d and 7 d treatment, are shown in Additional files 1, 2, 3 and Figures 1, 2, and 3.

### Morphology of shoot apices

Preliminary experiments showed that the shoot apices did not develop during the 3-week cold hardening period, therefore the initial development of flower primordia was monitored at the end of the 3-week recovery period, when the apices were isolated from the crowns of the seedlings under a Zeiss Stemi 2000-C stereomicroscope (Carl Zeiss Mikroskopie, Jena, Germany). The photos were taken with a Camedia digital camera using standardized exposure times and sensor settings. The photos of the apices are shown in Figure 8 and in Additional file 5. The developmental stages of the apices were determined based on the scale of Gardner et al. [51], which takes into account the appearance of new structures. The scale between 0 and 8 corresponds to the following developmental stages: 0 – vegetative apex, 1 – early elongation of the apex, 2 – elongation with single ridge, 3 – double ridge indicating the vegetative/generative transition, 4 – enlargement of spikelet primordia, 5 – empty glume primordia, 6 – lemma glume primordia, 7 – floret and anther primordia, 8 – terminal spike.

### Detection of peroxides

H_2_O_2_ was visualized in the shoot apex by staining with 10 μM 2′,7′-dichlorodihydrofluorescein diacetate (H_2_DCFDA) dissolved in 0.1 M Na-K-phosphate buffer (pH 8.0) for 30 min [52]. An Olympos BX 51 microscope (Olympos Optical Co. Ltd., Tokyo, Japan) fitted with a Camedia digital camera was used to study the stained shoot apices. The distribution of H_2_O_2_ in the apices is shown in Figure 8 and in Additional file 5.

### Gene expression studies

Total RNA was extracted with TRI Reagent (Sigma) according to the manufacturer’s instructions and the samples were treated with DNase I enzyme (Promega). Reverse transcription was performed using M-MLV Reverse Transcriptase and Oligo(dT) 15 primer (Promega) according to the manufacturer’s instructions. The expression level of the target genes was determined with real-time RT-PCR using a CFX96 thermocycler (Bio-Rad), with primers as detailed in Additional file 7[6,47,53-55]. The samples originated from 3 independent experiments each with 3 repetitions. The relative quantities of the individual transcripts were calculated with the ∆∆Ct method [56], using the housekeeping gene encoding a protein similar to phosphoglucanate dehydrogenase (unigene identifier: Ta307930) for normalization [54]. The gene expression value was set to 1 in control Ch plants and all other data were given as values relative to this in both genotypes in order to allow the two genotypes to be compared. The expression data are shown in Figures 5 and 6.

### Statistical analysis

Data from three independent experiments were evaluated, and standard deviations are indicated on the figures. The statistical analysis was done using two-component (treatments, genotypes) analysis of variance (SPSS program). Significant differences were calculated with the t-test. The correlation analysis was done according to Guilford [57].

## Abbreviations

ABA: Abscisic acid; APSR: Adenosine-5′-phosphosulphate reductase; ASA: Ascorbic acid; CAB: Calcium-binding protein; CBF14: C-repeat binding transcription factor 14; Ch: *Triticum ae.* ssp*. aestivum* cv. Cheyenne; COR14b: COLD-REGULATED14b; CyS: Cysteine; CySS: Cystine; DHA: Dehydroascorbate; ECys/CySS: Reduction potential of cysteine; EhmGSH/hmGSSG: Reduction potential of hydroxymethyl-glutathione; EGSH/GSSG: Reduction potential of glutathione; FKF1: FLAVIN-BINDING KELCH-REPEAT-BOX1 protein; GSH: Reduced glutathione; GSSG: Glutathione-disulphide; hmGSH: Reduced hydroxymethyl-glutathione; hmGSSG: Hydroxymethyl-glutathione disulphide; NCED1: 9-cis-epoxycarotenoid dioxygenase; NF-YB: Nuclear factor YB; OXS2: OXIDATIVE STRESS2; PEG: Polyethylene glycol; sAPX1: Ascorbate peroxidase (stroma); Tsp: *Triticum aestivum* ssp. *spelta*; VRN1: Major vernalization protein; ZCCT: ZINC-FINGER/CONSTANS, CONSTANS-LIKE, TOC1 domain flowering repressor protein.

## Competing interests

The authors declare that they have no competing interests.

## Authors’ contributions

KG and GG planned and supervised the study. ZG carried out the experiments and measured electrolyte leakage, growth parameters and gene expression. ÁB and AN examined the shoot apices. GS and PM measured the thiols by HPLC. All authors participated in data evaluation and the preparation of the manuscript. All authors read and approved the final manuscript.

## Supplementary Material

Additional file 1 Pharmacological modification of cysteine content and its reduction potential at optimal growth temperature. Click here for file

Additional file 2 Pharmacological modification of hydroxymethyl-glutathione content and its reduction potential at optimal growth temperature. Click here for file

Additional file 3 Pharmacological modification of glutathione content and its reduction potential at optimal growth temperature. Click here for file

Additional file 4 Effect of redox and osmotic treatments on the fresh weight of the shoots and roots of Ch (A) and Tsp (B) at optimal growth temperature. Click here for file

Additional file 5 Effect of redox and osmotic treatments on shoot apex morphology and peroxide content. Click here for file

Additional file 6 Correlation analysis of glutathione disulphide content, redox potential, gene expression, freezing tolerance and fresh weight. Click here for file

Additional file 7 Primers and program used for the determination of gene expression using real-time RT-PCR. Click here for file
