# Supplementary figures and images for: Central role of the flowering repressor ZCCT2 in the redox control of freezing tolerance and the initial development of flower primordia in wheat
Source: BMC Plant Biol. 2014 Apr 7;14:91. doi: 10.1186/1471-2229-14-91 (PMC4021066; doi:10.1186/1471-2229-14-91)

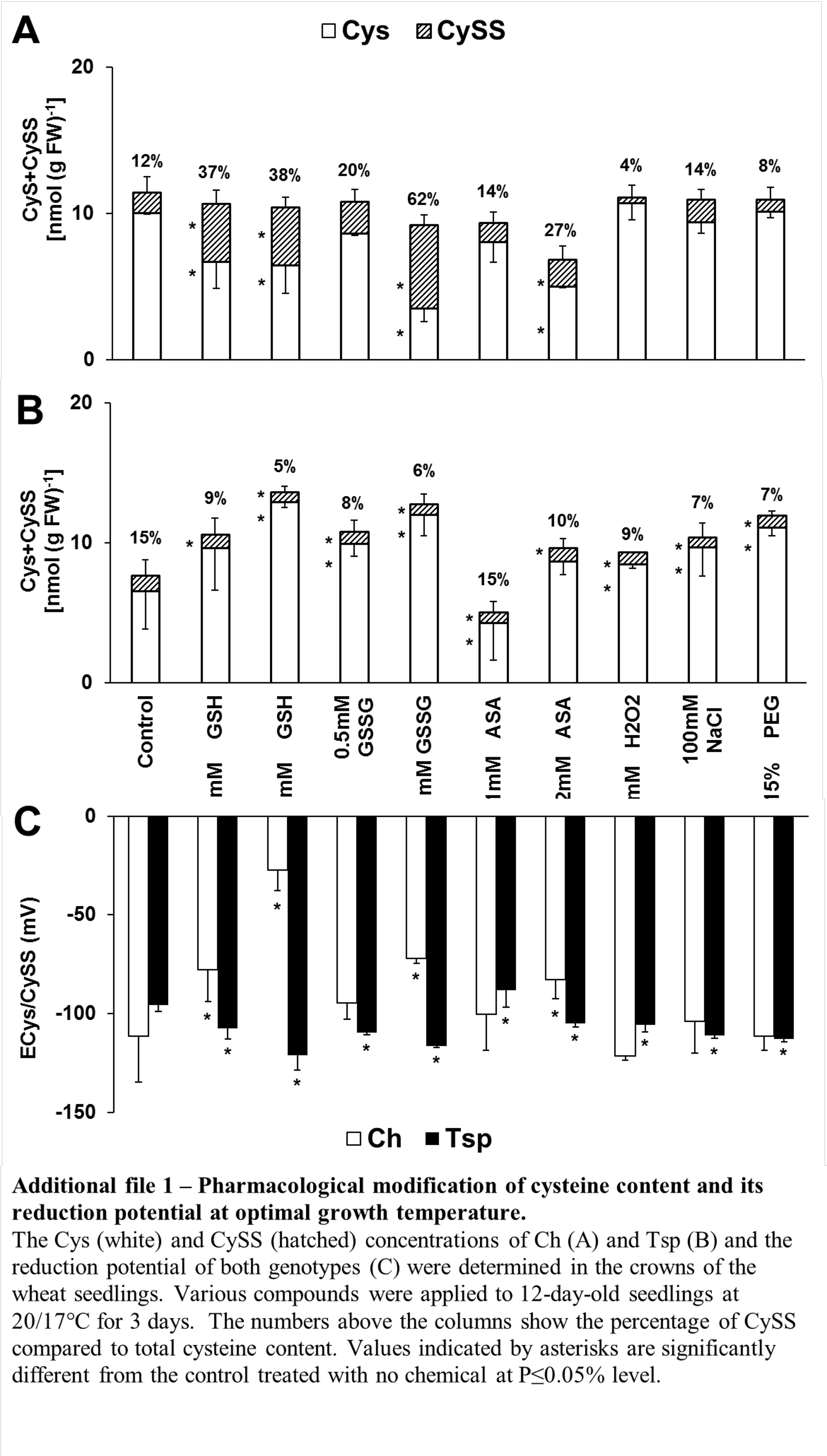

Supplement: Additional file 1 — Pharmacological modification of cysteine content and its reduction potential at optimal growth temperature. [file 1471-2229-14-91-S1.tiff]

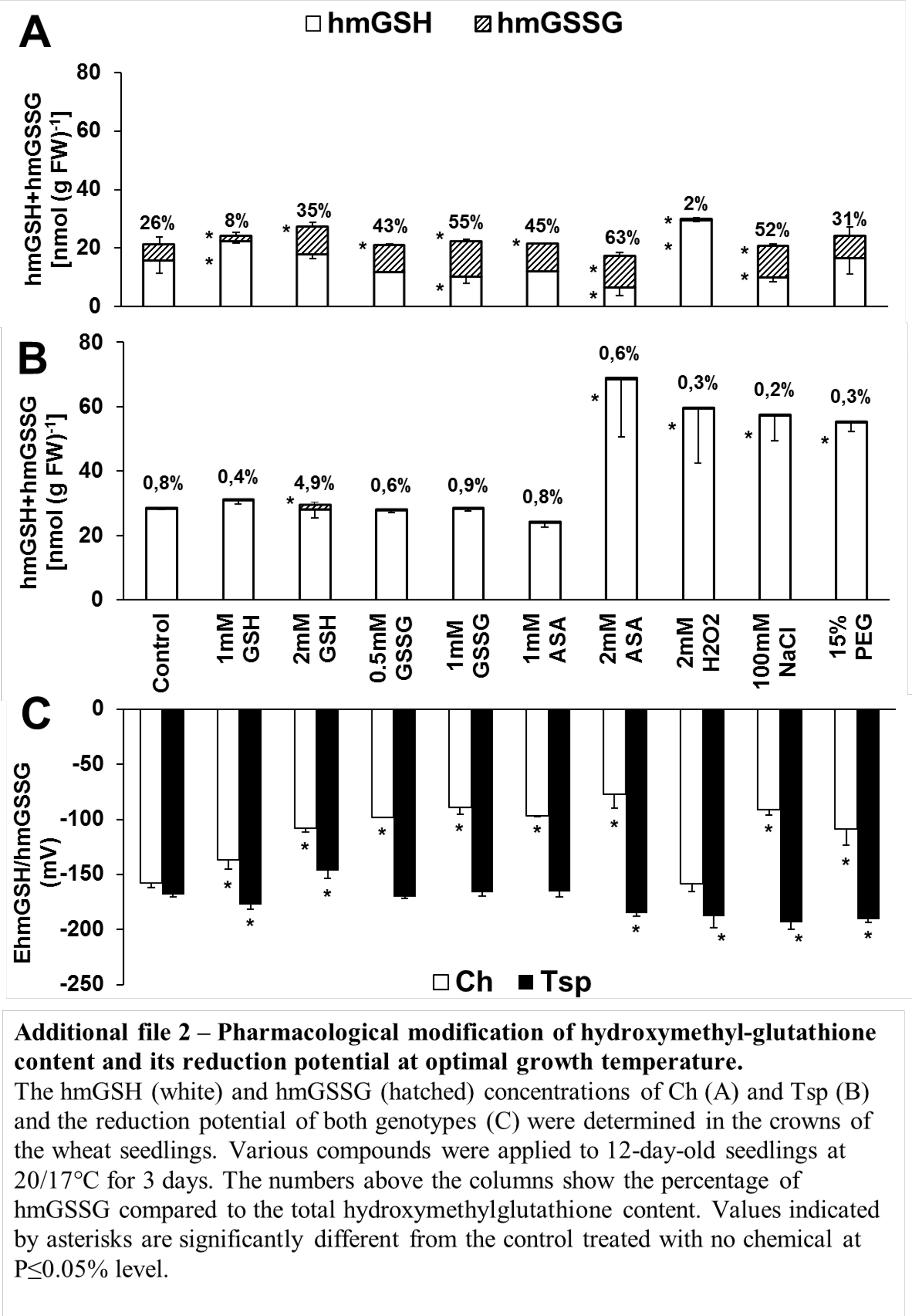

Supplement: Additional file 2 — Pharmacological modification of hydroxymethyl-glutathione content and its reduction potential at optimal growth temperature. [file 1471-2229-14-91-S2.tiff]

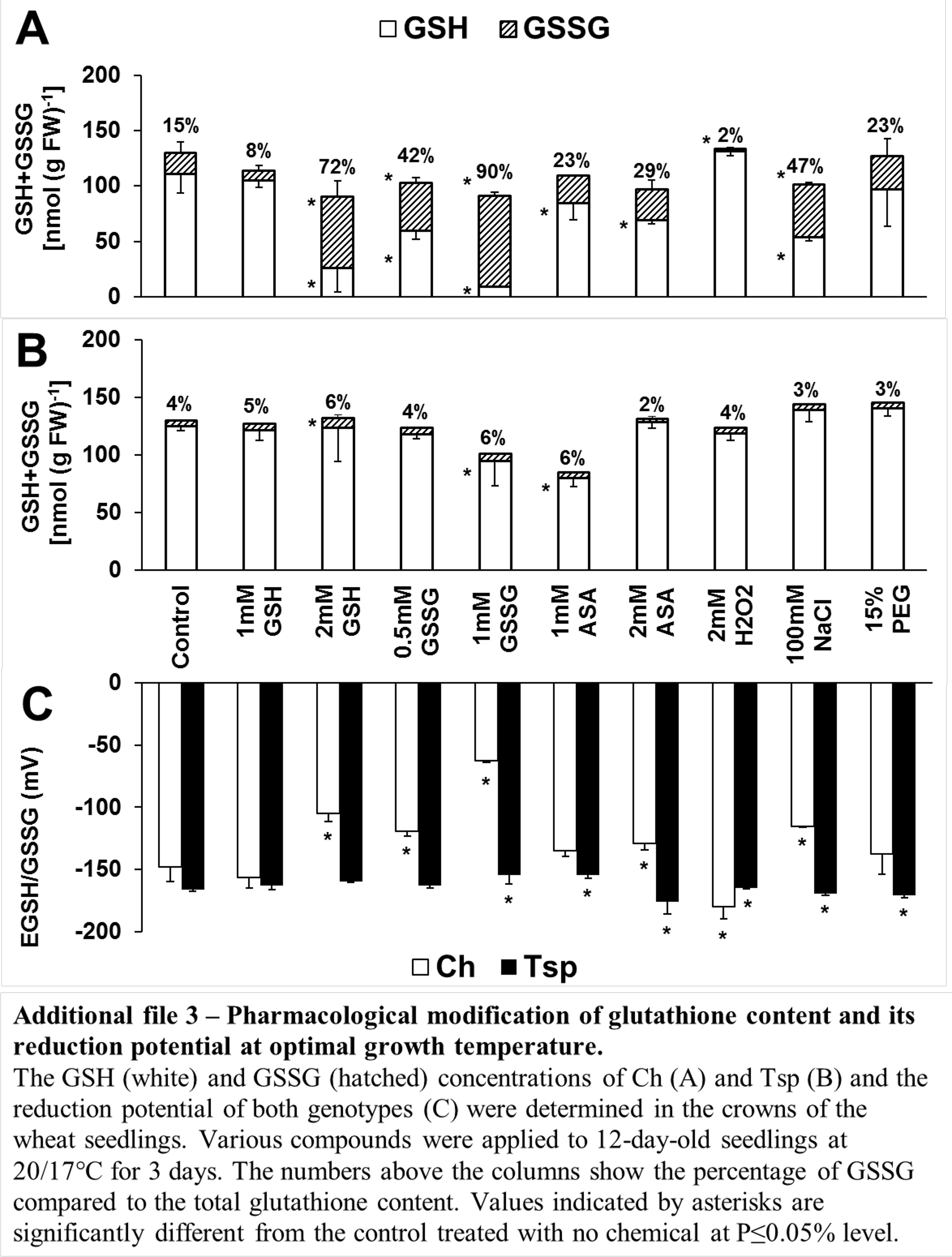

Supplement: Additional file 3 — Pharmacological modification of glutathione content and its reduction potential at optimal growth temperature. [file 1471-2229-14-91-S3.tiff]

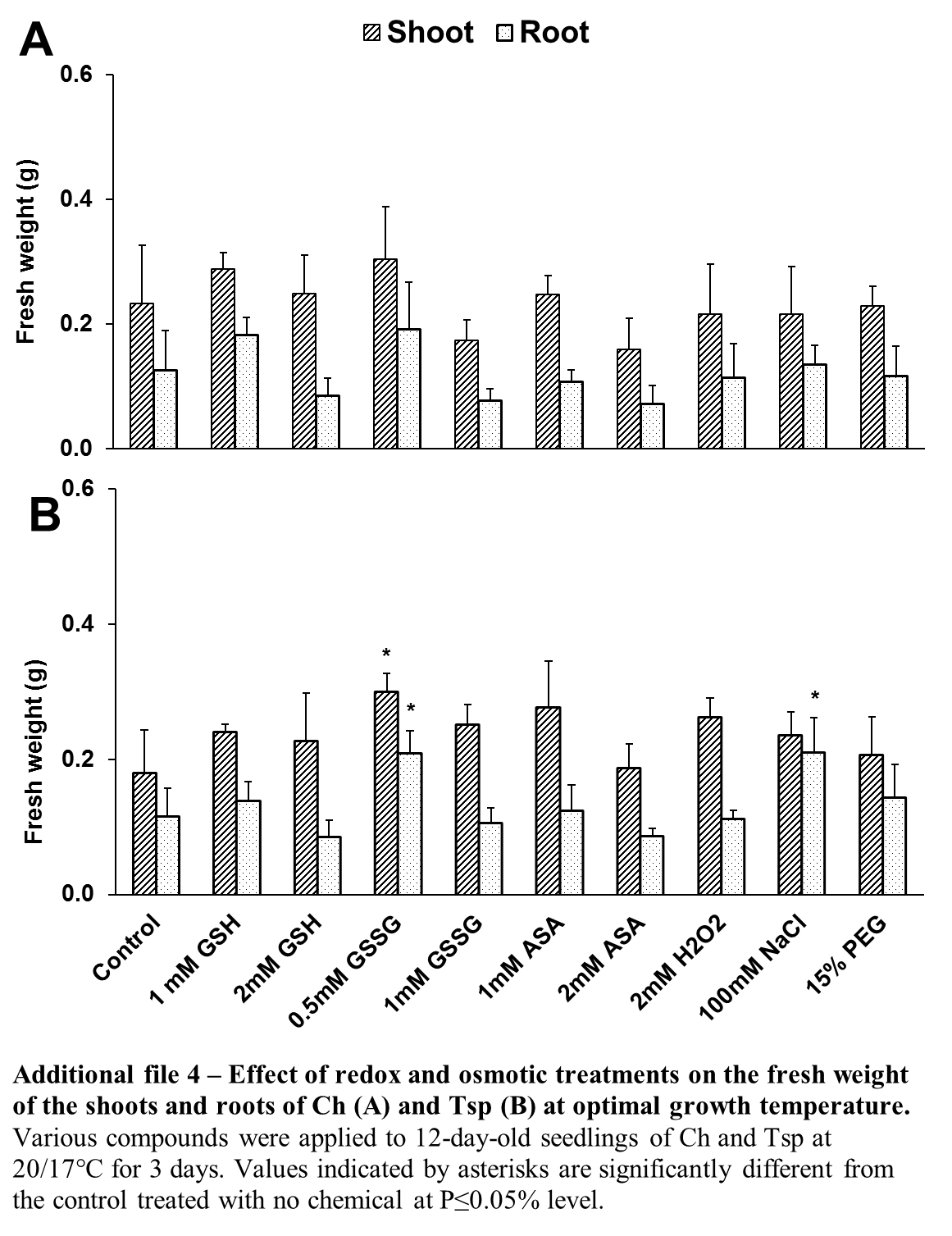

Supplement: Additional file 4 — Effect of redox and osmotic treatments on the fresh weight of the shoots and roots of Ch (A) and Tsp (B) at optimal growth temperature. [file 1471-2229-14-91-S4.tiff]

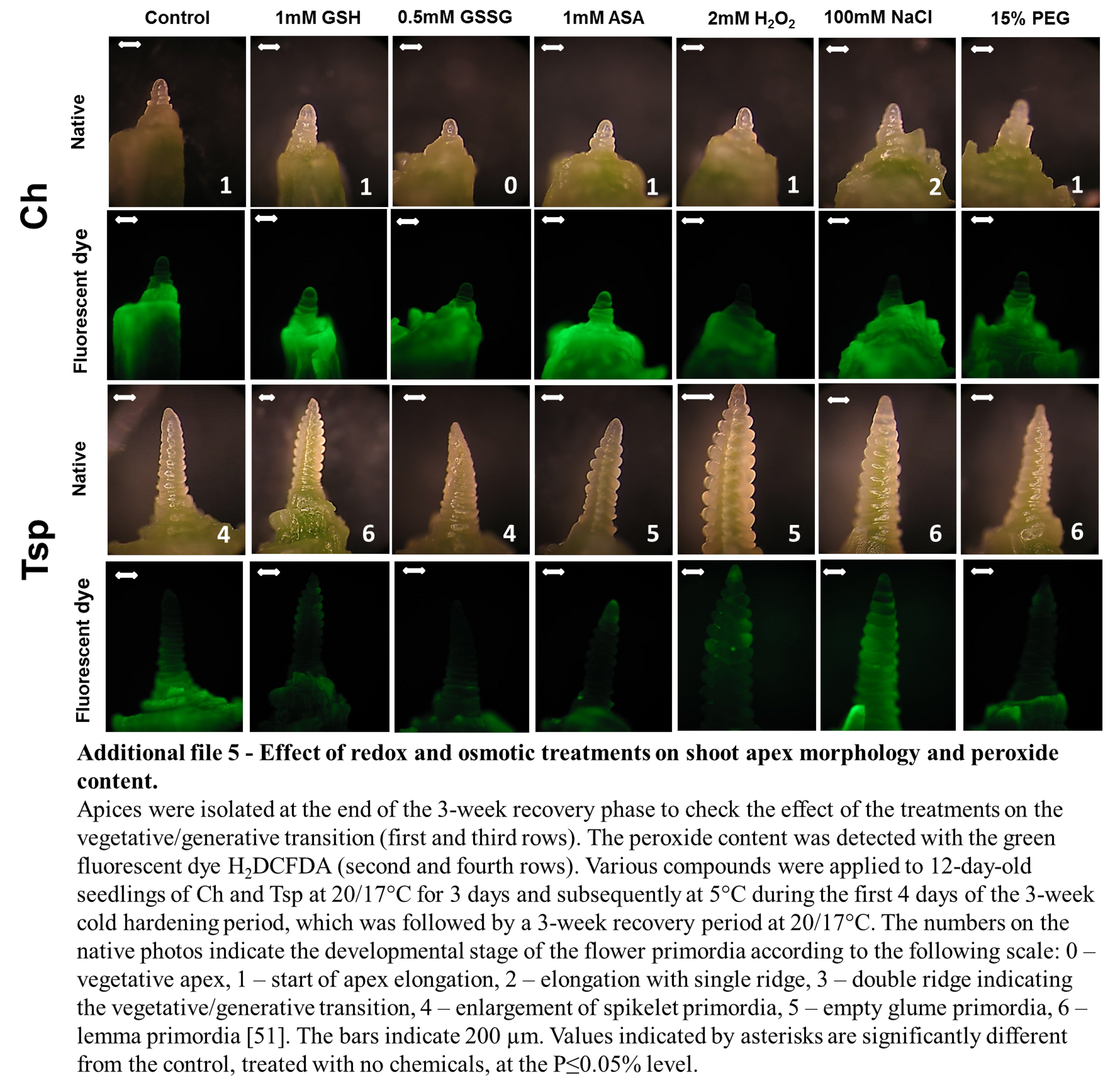

Supplement: Additional file 5 — Effect of redox and osmotic treatments on shoot apex morphology and peroxide content. [file 1471-2229-14-91-S5.tif]
